# Endometrial Cancer: Transitioning from Histology to Genomics

**DOI:** 10.3390/curroncol29020063

**Published:** 2022-01-31

**Authors:** Cristina Mitric, Marcus Q. Bernardini

**Affiliations:** 1 Department of Gynecologic Oncology, Princess Margaret Cancer Center, University Health Network, Sinai Health System, Toronto, ON M5B 2M9, Canada; cristina.mitric@uhn.ca; 2 Department of Obstetrics and Gynecology, University of Toronto, Toronto, ON M5G 1X8, Canada

**Keywords:** endometrial neoplasm, endometrial carcinoma, molecular classification, POLE, p53, mismatch repair, adjuvant treatment

## Abstract

Endometrial carcinoma (EC) is traditionally treated with surgery and adjuvant treatment depending on clinicopathological risk factors. The genomic analysis of EC in 2013 and subsequent studies using immunohistochemistry have led to the current EC molecular classification into: polymerase epsilon mutated (POLEmut), p53 abnormal (p53abn), mismatch repair deficient (MMRd), and no specific molecular profile (NSMP). The four groups have prognostic value and represent a promising tool for clinical decision-making regarding adjuvant treatment. Molecular classification was integrated into the recent European Society of Gynecologic Oncology (ESGO) management guidelines. POLEmut EC has favorable outcomes and retrospective studies found that omitting adjuvant treatment is safe in this group; two prospective clinical trials, PORTEC-4 and TAPER, are ongoing to assess this. p53 abn is associated with increased recurrence, decreased survival, and benefitted from chemotherapy in the PORTEC-3 subgroup molecular analysis. The clinical trials PORTEC-4a and CANSTAMP will prospectively assess this. MMRd and NSMP groups have intermediate prognosis and will likely continue to rely closely on clinicopathological features for adjuvant treatment decisions. In addition, the molecular classification has led to exploring novel treatments such as checkpoint inhibitors. Overall, the molecular perspective on EC and associated clinical trials will likely refine EC risk stratification to optimize care and avoid overtreatment.

## 1. Introduction

Endometrial cancer (EC) is the most common gynecological cancer, and the fourth most common malignancy in women in developed countries [1]. The lifetime incidence is 3%, and although most women present at an early stage and have a favorable prognosis, some women present with advanced disease, experience recurrences, and have poor prognosis [1]. The standard treatment for endometrial cancer is surgery involving total hysterectomy with bilateral salpingoophorectomy and lymph node assessment. The clinical and surgical histopathological features assist in classifying patients into risk categories to decide on need and type of adjuvant treatment. Adjuvant treatment can lead to significant toxicities such as cytopenias, gastrointestinal side effects, and quality of life consequences. As such, oncologists strive to achieve an optimal patient selection and provide an adequate balance between decreasing the risk of recurrence, optimizing survival, and avoiding side-effects associated with unnecessary overtreatment.

A recent important addition for achieving optimal patient selection for adjuvant treatment in the management of EC is the comprehensive genomic analysis by The Cancer Genome Atlas (TCGA) in 2013, dividing EC into four molecular subgroups based on mutational burden and copy number alterations [2]. The improved reproducibility of these classifications compared to historic histotyping make it an attractive strategy to incorporate in the everyday management of patients. The molecular classification was proven to have prognostic ramifications [3,4], however, there are limitations as to how this categorization affects subsequent treatment decision-making. The current review provides a summary of landmark trials defining traditional management in endometrial cancer, discusses the introduction of molecular classification, interprets trials in the context of the new molecular classification, and finally discusses current or proposed trials to define optimal treatment based on molecular classification.

## 2. Review of the Trials Defining Management in Endometrial Cancer

Traditionally, EC has been classified into low risk, intermediate risk (low-intermediate risk (LIR)/high-intermediate risk (HIR)), and high risk based on histopathological and clinical characteristics (Table 1). Specifically, poor prognostic factors such as histological type and grade, depth of myometrial involvement, presence of lymphovascular space invasion (LVSI), and patient age were used in the classification based on landmark trials GOG-99 and PORTEC [5,6]. Low-risk EC is unanimously defined as grade 1 or 2 endometrioid histology with less than 50% myometrial involvement [7]. Meanwhile, the definition of intermediate risk EC, specifically HIR EC, can be slightly different based on the reference trial [7]. Specifically, GOG group criteria are one, two, or three risk factors depending on whether the age of the patient is 70 and over, 50 to 69, or under 50, respectively [5]. The risk factors included in the definition are myometrial involvement >50%, grade 2 or 3 histology, and presence of LVSI. Meanwhile, the PORTEC definition is slightly different, requiring two out of three factors: age over 60, more than 50% myometrial involvement, or grade 3 histology [6]. The International Federation of Gynecology and Obstetrics (FIGO) guidelines combined the criteria of these landmark trials into a two out of five risk factors definition [8]. Patients who have poor prognostic factors that are not sufficient to meet HIR criteria are classified as LIR. Finally, the high risk group involves patients with high-risk histology, namely clear cell, serous type, patients with stage III or IV based on FIGO staging [8] as well as patients with deeply invasive grade 3 endometrioid [7,9].

The adjuvant treatment for EC based on the group is also illustrated in Table 1. For the low risk group, the management solely involves observations based on Danish data showing 93% survival at 68–92 months with high ability to salvage the vaginal recurrences [10]. For the intermediate group, GOG-99 evaluated the use of external beam radiation therapy (EBRT) and discovered that the use of radiation decreases the risk of recurrence, especially in the HIR group, with no statistically significant difference in overall survival [5]. Of note, occult stage II patients were included in this trial. Similarly, PORTEC-1 included patients with stage I EC and showed that the use of EBRT reduces locoregional recurrences with no statistically significant effect on survival [6], with 15-year long-term data confirming the relevance of HIR criteria for treatment selection, recommending against EBRT use for low risk and LIR patients [11]. Subsequently, PORTEC-2 randomized HIR patients to vaginal brachytherapy or EBRT in an open-label non-inferiority trial and showed similar recurrence and survival with less toxicity, thus advocating for brachytherapy use in this group [12].

For the high risk group, PORTEC-3 looked at the addition of chemotherapy to EBRT and showed an improvement in disease-free survival (DFS) for stage III patients, with no benefit for overall survival (OS) [13]. The GOG-258 randomized stage III and IVA patients into chemoradiation versus chemotherapy alone and found that chemoradiation was associated with less local and nodal recurrences, more distant recurrences, and no change in DFS [14]. GOG-249 included both HIR and high-risk patients in their selection, and randomized patients to either brachytherapy followed by three cycles of paclitaxel carboplatin or to EBRT. EBRT was associated with less nodal recurrence, less short-term complications, and similar survival, concluding that EBRT should remain the treatment of choice for high risk EC [15]. The included patients and results of these landmark trials are summarized in Table 2. In terms of future research, there is currently a randomized phase II trial Danish trial (ENGOT-EN2-DGCG trial) comparing chemotherapy with observations in patients with stage I or II EC, known negative lymph nodes, and fitting HIR or high risk criteria. The trial includes optional brachytherapy in both arms and is anticipated to be completed in January 2023 [16].

Although these landmark trials are prospective studies, several aspects limit the ability to draw conclusions on the optimal management based on clinicopathological factors alone. First, the studies had overlapping cohorts, for instance, there were differences between HIR definitions in the GOG and PORTEC trials such as clear cell and serous EC being included in PORTEC-1, but not in GOG-99 [5,6]. Not only were the risk designations different between trials, but the definitions have evolved over the last two decades [9]. Furthermore, surgical staging was inconsistent in terms of lymph node assessment requirements. Finally, none of the landmark trials described above were sufficiently powered to analyze the differences between histological subtypes. Although histology plays a role in determining prognosis and adjuvant treatment, classifying patients into type I EC if there is a grade 1 and 2 endometrioid, or type II EC if a grade 3 or non-endometrioid histology does not fully explain the clinical picture. Indeed, the CHREC (Canadian High risk Endometrial Cancer Consortium) cohort study included 1260 patients with type II EC from seven institutions and showed that there was variation in response to treatment, for instance, grade 3 endometrioid EC and clear cell EC showed improved OS with adjuvant radiation whereas serous subtype EC and carcinosarcoma showed improved OS with adjuvant chemotherapy [17]. As such, combining non-endometrioid histologies under the high-risk EC subgroup may have an insufficient value for choosing an adequate adjuvant treatment. In addition, this Canadian study raised the issue of inter-observer variability when using clinicopathological factors alone to guide management as it found significant differences between the institutions with respect to the use of adjuvant chemotherapy or radiation [17].

## 3. Introduction of Molecular Classification

**Figure 1:**
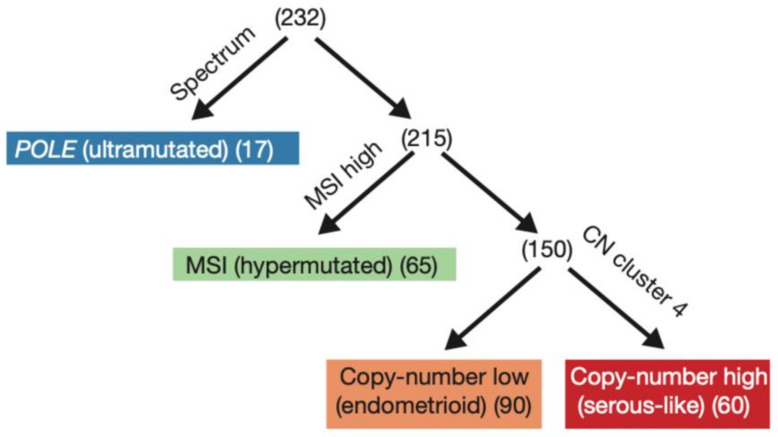
The TCGA (The Cancer Genome Atlas) 2013 original classification. Reprinted with permission from “Integrated genomic characterization of endometrial carcinoma” by Levine et al. [2]. Molecular classification into the four groups by (1) nucleotide substitution frequencies and patters, (2) MSI status, and (3) copy-number cluster; POLE polymerase epsilon, MSI microsatellite instability, CN copy number.

**Figure 2:**
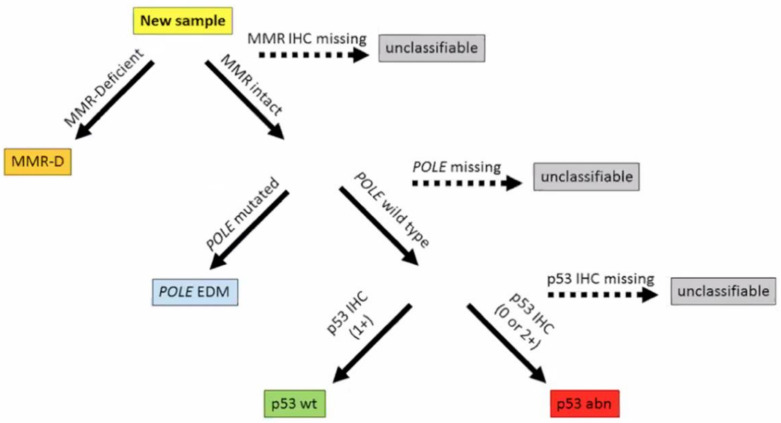
The ProMisE (Proactive Molecular Risk Classifier for Endometrial Cancer) original molecular classification. Reprinted with permission from “Confirmation of ProMisE: A simple, genomics-based clinical classifier for endometrial cancer” [19]. MMR-D mismatch repair deficiency, POLE EDM polymerase epsilon exonuclease domain mutation, IHC immunohistochemistry, wt wild type, abn abnormal. p53 IHC intensity as absent (0) or overexpressed (+2) is classified as p53 abn, whereas some level of p53 IHC expression (+1) is interpreted as p53 wt (19).

The 2013 comprehensive genomic analysis of EC by The Cancer Genome Atlas (TCGA) divided 373 cases of serous and endometrioid EC into four molecular subgroups based on mutational burden and copy number alterations: precisely POLE mutated, microsatellite instability (MSI), copy-number low, and copy-number high (Figure 1) [2]. The study also demonstrated that the four categories were different in terms of prognostic outcomes, with 5-year PFS being best with POLE mutated ECs and worst with copy-number high ECs [2,4]. Prognostic information provided by genomic analysis was similar to the prognostic value of histologic classification. For instance, survival curves were similar between high copy-number and serous subtype, and between the other clusters and endometrioid histology; however, the other clusters were differentiated into three groups with POLE mutated having the best survival prognosis, and MSI and copy-number low having an intermediate prognosis. It was also found that POLE mutated tumors are often associated with the endometrioid EC subtype, whereas copy-number high consists of serous and high-grade endometrioid EC [2]. In a molecular analysis of the PORTEC 1 and 2 cohorts, 97% of tumors were molecularly classifiable, and the classification improved the prognostic ability of clinicopathological features in the same cohort [4]. Subsequently, two groups used a combination of focused sequencing of POLE and immunohistochemistry (IHC) to create and validate surrogate categories, representing a more easily achievable classification (Figure 2) as it can be performed on standard formalin-fixed, paraffin-embedded material [18,19,20]. This classification divided EC tumors into categories based on: POLE mutation status, MMR status, and p53 status, in this precise order [18,19].

Tumors where testing was not carried out or was inconclusive were deemed unclassifiable, whereas tumors where all the tests were negative were named as “no specific mutation present” (NSMP). Approximately 3–6% of tumors have characteristics pertaining to two classification criteria, and these tumors are termed multiple-classifier EC [21]. The initial studies showed a stronger incidence of TP53 mutations in the serous histologic subtype and high grade endometrioid, with infrequent TP53 mutations and more common POLE mutations in the low grade endometrioid histology tumors [2]. Subsequent studies looking at this molecular division showed that the four groups can be found across all stages, histologic types, and grades [22].

Furthermore, the molecular classification provides an ability to sort tumors that is standardized, highly reproducible, and shows concordance between the initial biopsy and final hysterectomy specimen. These are all advantages compared to historical histological classification [3,23,24]. In particular, the concordance between the initial biopsy and the final hysterectomy specimen has important future implications for pre-operative planning, for example, leading to potentially omitting sentinel lymph node dissection in POLE-mutated biopsies, or perhaps completing full lymphadenectomy in p53 abn tumors on endometrial biopsy.

### 3.1. POLE Ultramutation

The POLE mutation is present in 6–9% of EC specimens and presents in younger, thinner women, often associated with the endometrioid histological subtype and early stage [2,25]. It is a mutation in the exonuclease domain of POLE, a gene coding for DNA polymerase epsilon, which is involved in DNA replication and repair [2]. This defect results in an ultra-mutated tumor, which is believed to lead to increased immune response against mutated cells, as it has been shown to result in increased CD8 lymphocytic infiltrate and upregulation of cytotoxic T-cells [26]. POLE-mutated tumors often show aggressive pathologic features such as high grade or LVSI, but nonetheless result in favorable prognosis with 96% survival at five years [2]. A subsequent study in the PORTEC cohorts confirmed a very favorable prognosis regardless of the adjuvant therapy [4]. In a study looking at clinicopathological and molecular characterization of multiple-classifier EC, patients having both POLE ultramutation and abnormal p53 status were found to have molecular clustering and clinical outcomes similar to EC solely characterized by POLE mutation [21]. While these findings were limited by the small number of samples, the study did generate the hypothesis of wheter these EC tumors should be classified in the POLE category [21]. This should be carefully decided only after confirming that the POLE mutation is indeed pathogenic, as many mutations in this gene are not pathogenic and could lead to a false interpretation [27].

In terms of treatment, a recent meta-analysis looking at the use of adjuvant treatment for POLE-mutated tumors showed no additional survival benefits with therapy, independent of the adjuvant treatment used [28]. As such, prospective trials looking at de-escalating care in these patients are ongoing and discussed in the last section of the current review [29]. De-escalating care appears to be a safe step based on a recent meta-analysis, with recurrences in only 11 out of 294 patients (3.7%), along with a high salvage rate and good survival in eight out of the 11 patients (72.7%). Additionally, given the resulting immunity upregulation in POLE-mutated tumors, immune checkpoint inhibitors are a potential treatment to use [3].

### 3.2. P53 Abnormal

The Cancer Genome Atlas initially established the category of copy-number high, which correlated with serous and high grade endometrioid EC as well as TP53 mutation [2]. Furthermore, there were molecular similarities between the copy-number high EC and high-grade serous tubo-ovarian cancer and basal-like breast carcinoma, with all groups being characterized by TP53 mutations as well as low rates of PTEN mutations [2,30]. Later, the ProMisE trial found the p53 abnormal IHC to be an adequate surrogate for the copy-number high molecular group, and these tumors represent 13–18% of all EC [19]. There was a strong correlation between p53 abnormal IHC finding and TP53 mutation obtained by sequencing, with reliable inter-laboratory reproducibility when tested on endometrial biopsy samples [31]. The p53 category is demographically described as associated with older age and lower body mass index (BMI), and clinically as associated with more advanced stage as well as poorer prognosis, being responsible for 50–70% of EC mortality [19,30]. From a histological perspective, the proportion of p53 abnormal was found to be 93% in serous EC, 85% in carcinosarcoma, 38% in clear cell EC, 22% in grade 3 endometrioid EC, and only 5% in grade 1 and 2 endometrioid EC [30]. In each of these histologic subgroups, it was associated with a worse prognosis [30]. When molecular categorization finds an abnormal p53 status in addition to MMR deficiency or POLE mutation, the tumor is placed in the MMRd or POLE mutation, respectively. This is due to a study showing clustering and similar prognosis of multiple classifiers to the MMRd and POLE groups respectively, hypothesizing that the TP53 mutation is a later event during tumor progression in MMRd or POLE mutant tumors [21].

In the molecular-based analysis of the PORTEC-3 cohort, EC patients with abnormal p53 were found to have improved outcomes when platinum-based chemotherapy was added to the adjuvant radiation [32]. Specifically, the 5-year RFS was 59% with chemoradiation versus 36% for radiation alone (*p* = 0.019) [32]. The use of radiation in the p53 abnormal group thus becomes questionable [30]. A potential avenue for treatment of p53 abnormal EC could be targets looking at HER-2 (human epidermal growth factor receptor 2) and HRD (homologous recombination deficiency) as abnormal p53 tumors were found to be correlated with HER2 status in an analysis of the PORTEC-3 cohort and with HRD in another study [33,34]. A small, randomized phase II clinical trial looked at transtuzumab, which is the main monoclonal antibody drug that targets HER2, as an adjuvant treatment to standard chemotherapy during treatment and as maintenance in patients with advanced and recurrent serous EC, and found a PFS benefit of 17.9 months versus 9.3 months and an OS benefit of more than five months in the advanced setting [35,36]. There are, however, no other studies looking at transtuzumab based on molecular classifiers such as abnormal p53. Nonetheless, the SGO included, in their recent 2021 guidelines, the recommendation of IHC Her2Neu testing for patients with stage III or IV serous EC [9]. As for the HRD association, PARP (poly adenosine diphosphate-ribose polymerase) inhibitors represent potential treatments that have not yet been explored in clinical trials but will be part of future trials, as discussed below. Finally, an exploratory analysis looked at adding bevacizumab versus temsirolimus to standard chemotherapy in advanced EC, and found that TP53 mutation was associated with improved survival outcomes in the bevacizumab subgroup [37].

### 3.3. MMR Deficient (MMRd)

The MMR deficient molecular group represents 20–30% of EC cases, and is analogous to MSI in the initial genomic classification [2,19]. Tumors that are MMRd or MSI-high can originate through three pathways: germline MMR mutations in DNA mismatch repair proteins MLH1, PMS2, MSH2, MSH6, named Lynch syndrome; somatic MMR gene mutations occasionally labelled as Lynch-like; and homozygous methylation of the MLH1 gene promoter named sporadic [38]. These mutations are detected by IHC, making them less costly to obtain, and as such, this histological step has been implemented in many institutions. The initial role of this implementation was to assist with confirming histological diagnosis and to screen for Lynch syndrome, which represents approximately 3% of EC [18,39]. More recently, knowing the MMR status of an EC tumor also has prognostic value and determines potential access to newer treatments such as checkpoint inhibitors [9,40]. The MMR status has been found to be associated with an intermediate prognosis for EC [19], and more commonly associated with endometrioid EC [41]. From a demographic perspective, there was no age group or BMI association with MMRd [25].

In terms of molecularly directed adjuvant treatments, recent retrospective studies have raised the hypothesis that MMRd ECs might have a stronger susceptibility to radiation with improved survival compared to MMR proficient tumors, however, this has not been studied prospectively [41,42,43]. Checkpoint inhibitors pembrolizumab and lenvatinib were shown to have beneficial effects and are currently part of the recommended systemic second-line treatment for MMR proficient tumors in the NCCN’s (National Comprehensive Cancer Network) most recent guidelines [40]. These medications showed encouraging results and received an accelerated approval for MMR proficient solid tumors including EC [44,45]. The approval was a collaborative international review involving the Food and Drug Administration (FDA), Health Canada, and the Australian Therapeutic goods Administration, leading to a simultaneous approval decision in all three countries [45]. These benefits were reconfirmed in a recently published phase 3 trial: the combination of pembrolizumab and lenvatinib were shown to improve both OS and PFS when compared to second or subsequent line chemotherapy in MMR proficient patients [46]. Additionally, pembrolizumab alone is also FDA approved for EC and recommended for advanced or recurrent MMRd rather than proficient tumors in the recent NCCN guidelines [40,47]. Other immune checkpoint inhibitors investigated in the context of MMRd tumors are dostarlimab and durvalumab. Dostarlimab is a humanized programmed death (PD)-1 receptor monoclonal antibody that blocks interaction with the PD-1 ligands and was the key treatment for the recent GARNET trial. This multi-center open-label phase I/II trial was designed to assess the clinical activity and safety of dostarlimab in patients that received two or less prior lines of treatment for advanced or recurrent EC. The interim analysis recently presented showed the rate of the objective response rate (ORR) to be 45% with 11% complete response and 34% partial response [48], leading to FDA approval for EC [47]. Durvalumab is another antibody to PDL1 that has shown some promising results in patients with MMRd advanced EC, with a similar ORR [49]. Avelumab was also studied in advanced or recurrent EC based on MMR status and a more modest ORR finding of 27% was found for the MMRd group and no benefit for the MMR proficient patients [50]. Additionally, the clinical trial NCT02912572 is currently recruiting patients to look at the use of avelumab in combination with either the PARP inhibitor talazoparib or the tyrosine kinase axitnib in patients with EC based on MMR status and is expected to be completed in 2022 [51]. A recent trial by Bellone et al. found that the benefit of using checkpoint inhibitors in MMRd tumors seems to be driven by the effect on Lynch and Lynch-like tumors rather than the sporadic ones [38].

### 3.4. P53wt/No Specific Mutation Profile (NSMP)

The NSMP profile is p53 wild type, MMR proficient, shows no POLE mutation, and has been found to be associated with 40–50% of ECs [19]. This molecular group corresponds to the copy-number low in the initial genomic classification and was found to have an intermediate prognostic value [2,19]. From a demographic perspective, patients in this subgroup have the highest BMI [25]. This group mostly includes endometrioid ECs with estrogen and progesterone receptor (ER, PR) positivity and high response rates to hormonal therapy [18]. Attempts to further categorize the NSMP ECs found it to be associated with the CTNNB1 (beta catenin 1) and L1 cell adhesion molecule (L1CAM) mutation, which confines them to a poorer prognosis. CTNNB1 mutation has been found to be associated with more distant recurrence in the PORTEC cohorts [4,52]. In a separate study looking at low-grade early stage endometrioid EC, CTNNB1 mutation was also shown to be associated with worse recurrence-free survival [53]. L1CAM is a membrane glycoprotein that plays a role in tumor cell migration. It is a strong predictor of decreased survival and is associated with adverse clinicopathological characteristics, namely >50% myometrial invasion, LVSI, and lymph node involvement [54]. L1CAM has also been found to assist with the classification of patients that were otherwise unclassified within the traditional EC molecular subgroup classification and is strongly associated with p53 [55]. They are both included in ongoing EC clinical trials such as PORTEC-4 [52].

NSMP tumors have also been found to be associated with mutations in the PI3K/Akt/mTOR pathway and given their association with positive estrogen and progesterone receptors (ER+ PR+), a few studies have looked at treatments targeting these receptors. The use of eeverolimus and letrozole in a phase II study on recurrent EC showed an ORR of 32% [56]. Another study on the cyclin-dependent kinase inhibitor palbociclib combined with letrozole showed a disease control rate of 64% compared to letrozole alone and improved PFS by five months [57]. However, none of these studies were stratified by molecular subtype. Given the lack of specific molecular markers in the NSMP group and limitations of clinical studies in this category, the adjuvant treatment will likely continue to rely significantly on clinicopathological criteria [3].

## 4. Interpreting Trial Data in the Context of New Molecular Classification

As the introduction of molecular categorization in EC is adding new information regarding the risk of recurrence and survival, a likely transition phase in the treatment of EC will involve refining the clinicopathological risk groups with the addition of molecular risk factors. However, to date, large prospective studies have not been performed to confirm this hypothesis. Nonetheless, in 2020, the European Society of Gynecological Oncology (ESGO), European Society for Radiotherapy and Oncology (ESTRO), and European Society of Pathology (ESP) updated their management guidelines by combining molecular and clinicopathological factors in the risk stratification [39], while the Society of Gynecologic Oncology (SGO) 2021 guidelines did not [9]. Specifically, they re-assigned the low, LIR, HIR, and high EC groups when the molecular classification is known (Table 1). The presence of POLE mutation re-classified all stage I and II tumors into the low-risk category. For advanced POLE mutation tumors, there are no recommendations regarding classification given the limited evidence [39]. The p53 abnormal status upgraded the category of most stage I–IVA tumors to the high risk group, with the exception of stage IA with no myometrial invasion, which was assigned to LIR. Given the intermediate prognosis of MMRd and NSMP tumors, they were classified according to concomitant clinicopathological features such as presence of myometrial invasion, LVSI, high-grade histology, or stage [39]. In particular, clear cell carcinoma has not been classified given the limited data [39]. A retrospective study using the ESGO 2020 guidelines to re-classify 594 patients including molecular characteristics led to a change in group assignment in 39 patients (7%): 17/39 (44%) were upgraded due to p53 abnormal IHC, and 22/39 (56%) were downgraded due to POLE mutation [58]. The reference in this study was the ESGO 2016 classification, which differed slightly from the North American risk stratification. Of note, the guidelines draw attention to the fact that implementation of molecular features into EC risk stratification can only be performed if there is simultaneous assessment of p53, MMR, and POLE status, given the presence of multiple molecular classifiers in 3–6% of tumors [21,39].

The changes in treatment brought along by the introduction of molecular classification have implications on the choice of adjuvant treatment given changes in risk stratification as well as on the choice of novel molecular-based therapies. First, once the molecular characteristics are integrated into a refined risk group classification of EC, the selection of adjuvant treatment would follow the specific risk group assigned adjuvant therapy, as previously established. For low-risk and LIR EC, no adjuvant treatment is required. When molecular classification is known, stage I–II patients with POLE mutation are to omit adjuvant treatment in the new ESGO guidelines [39]. This is supported by the lack of benefit of adjuvant therapy described in a recent meta-analysis [28]. The ESGO guidelines do not include more advanced stage (III–IVA) POLE mutated patients in this category given the data limitation [39]. The ESGO guidelines also suggest potentially omitting POLE-mutation analysis in low-resource setting for patients classified as low risk and LIR EC with grade 1 or 2 endometrioid, with a recommendation graded as IV C [39]. Indeed, for patients not requiring adjuvant treatment based on clinicopathological factors, knowing the POLE mutation status would not impact management. The HIR category has been least impacted by the introduction of molecular features and continues to rely greatly on clinicopathological factors [39]; the adjuvant treatment is brachytherapy to decrease vaginal recurrence, as described previously. Tumors reclassified in the high risk category by the presence of abnormal p53 will benefit from the addition of chemotherapy, and this is supported by the observed benefit in the PORTEC-3 cohort analysis [32]. An exception related to p53 abnormal status are tumors restricted to a polyp or without myometrial invasion, where the need for adjuvant treatment is not recommended in the ESGO guidelines [39]. Second, the knowledge produced by the molecular classification provides information on the underlying tumor replication mechanism and represents a potential target of treatment. Thus far, the only molecular-based therapies approved by FDA for EC are pembrolizumab and dostarlimab as stand-alone therapies for MMRd tumors, and the combination of pembrolizumab and lenvatinib for MMR proficient tumors, both as second line systemic treatments [39,45,47]. So far, all studies analyzed novel treatments in the advanced or recurrent EC setting [39]. Prospective studies such as RAINBO, CANSTAMP, NRG-GY018, and GY020, which are described in the next section, are the first studies to analyze molecular-based treatments in the primary setting.

## 5. Current and Proposed Trials to Define Optimal Treatment Based on Molecular Classification

**Figure 3:**
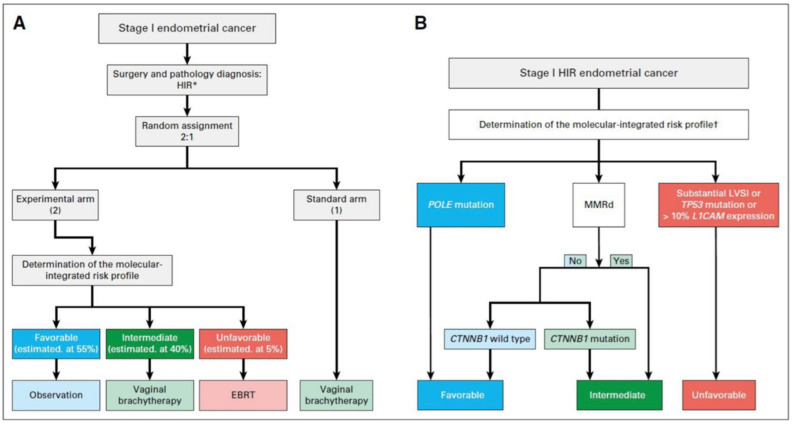
Study design of the PORTEC-4a trial. Reprinted with permission from “PORTEC-4a: international randomized trial of molecular profile-based adjuvant treatment for women with high-intermediate risk endometrial cancer [52]. HIR high intermediate risk, EBRT external beam radiation therapy, POLE polymerase epsilon, MMRd mismatch repair deficiency, LVSI lymphovascular space invasion, L1CAM L1 cell adhesion molecule, CTNNB1 beta catenin 1. (**A**) Study design of the PORTEC-4a trial. (**B**) Decision tree of the molecular-integrated risk profile.

So far, the evidence regarding adjuvant treatment of EC based on molecular classification is limited to retrospective studies and secondary molecular analyses of patients receiving standard adjuvant treatment. There are several clinical trials currently looking at individualizing treatment based on molecular characteristics (Table 3), of which two of these are phase III trials. The first one is PORTEC-4a, focusing on HIR EC randomizing patients 1:2 to a standard treatment with vaginal brachytherapy, and an experimental group where they received treatment based on their molecular profile, being classified as favorable (POLE mutation or CTNNB1 wild type), intermediate (MMRd or CTNNB1 mutated), or unfavorable (LVSI, TP53, or L1-cell adhesion molecule L1-CAM overexpression) [52]. Specifically, the favorable profile will receive observation alone, the intermediate profile will receive vaginal brachytherapy, and the unfavorable profile will receive EBRT [52]. Of note, the L1CAM, CTNNB1, and LVSI clinicopathological criteria were added as they are risk factors for local and distant recurrences [52]. The primary outcome is vaginal recurrence at five years, and the secondary outcomes include OS, RFS, quality of life questionnaires, adverse events, and health care costs. The study design and treatment plan are illustrated in Figure 3 [59]. Initial evaluation of the pilot phase found the study to be feasible with a satisfactory patient acceptance rate and an average time between randomization and the determination of the molecular-integrated risk profile of 10.2 days [59]. PORTEC-4a will be the first trial to prospectively investigate the use of adjuvant therapy after combining molecular and clinicopathological features in EC. The protocol development is ongoing for the second trial, the RAINBO umbrella program (Refining Adjuvant treatment iN endometrial cancer Based On molecular profile), which is a TransPORTEC international collaboration of personalized molecular-based adjuvant treatment for patients with high risk EC (Table 4) [60]. The multi-disciplinary program involves the Danish, French, Netherland, American, Canadian, and Australian New Zealand groups (DGOG, GINECO, NCRI, CCTG, ANZGOG). The recruited high-risk EC patients will be centrally registered and classified into one of the four sub-trials: p53 abnormal, MMRd, POLE mutated, and NSMP. The decision for the treatment arms is based on the PORTEC-3 analysis using molecular classification [32]. Specifically, the POLE mutated patients will receive no adjuvant treatment, while the p53 abnormal patients will be randomized to chemoradiation or chemoradiation with PARP inhibitor. The intermediate prognosis group MMRd will be randomized to radiotherapy alone or radiotherapy with a checkpoint inhibitor, and the NSMP patients will be randomized to chemoradiation or radiation with hormonal treatment. The primary endpoint will be 5-year RFS [60]. The RAINBO and PORTEC-4a molecular studies are expected to provide a clinical decision-making tool for adjuvant treatment of patients with HIR and high risk EC to increase RFS with less toxicity.

Two additional Canadian-based studies are looking at specific molecular subtypes, namely, POLE and p53 status. The trial entitled TAPER (Tailored Adjuvant therapy in POLE-mutated and p53 wildtype early-stage Endometrial canceR) is a multi-center, single-arm prospective cohort study that will look specifically at de-escalating care in patients with POLE-mutated and p53-wildtype (NSMP) early stage endometrioid EC. The MMRd and p53 abnormal patients will be excluded. Included patients will receive no treatment postoperatively, in order to prospectively confirm the safety of omitting radiotherapy in POLE mutated and p53 wild type EC, as has been retrospectively described [28]. The primary endpoint will be the probability of pelvic recurrences (including vaginal recurrences) at three years [29]. The assumption is that 3-year pelvic relapses will be at less than 4%, and the trial will be halted should this limit be exceeded. The secondary outcomes will include 3-year RFS and OS, sites of relapse assessment, and patient decision making data analysis. This study is currently recruiting and has an estimation completion date in December 2023 [29]. The second Canadian study CANSTAMP (NCT04159155) is a multi-arm, multi-stage randomized controlled trial assessing front line treatment in serous or p53 mutated EC [61]. The study is divided into three cohorts: early stage, advanced stage, and exploratory cohort. The cohorts will have different arms analyzing different treatment effects. The early-stage cohort and will compare chemotherapy (carboplatin and paclitaxel) alone with the combination of chemotherapy and chemoradiation. The primary outcome is PFS at three years and the secondary outcome is OS and number of adverse events at five years. The second cohort will include patients with stage III and IV EC serous or p53 mutant, and the treatment arms will compare chemotherapy versus chemotherapy with the PARP inhibitor niraparib. The estimated completion date is September 2025 [61]. The exploratory arm will include patients with p53 abnormal or serous cancers that do not qualify for the other cohorts.

There are currently two phase III trials recruiting patients to assess the effect of pembrolizumab’s addition to standard treatment for MMRd EC. The NRG-GY018 (NCT03914612) is a randomized quadruple blinded trial (patient, care provider, investigator, outcome assessor) looking at the benefit of adding pembrolizumab to standard chemotherapy with carboplatin and paclitaxel [62]. The control group undergoes chemotherapy with the placebo. The patients included have stage III, IV, or recurrent EC and have known MMR IHC status. The IHC is assessed at the institutional level and there is a subsequent centralized quality control. The primary endpoint is PFS at five years and secondary endpoints include objective tumor response, duration of objective response, OS as well as adverse events, quality of life, and patient-reported outcomes (PROs). This study represents a collaboration between the Canadian Cancer Trials Group and NRG Oncology and has an estimated completion date of June 2023 [62]. The second study NRG-GY020 (NCT04214067) assesses pembrolizumab in patients with HIR EC that are MMRd by using the GOG HIR criteria. The patients undergo 2:1 randomization to radiotherapy and pembrolizumab as maintenance for one year versus radiotherapy alone postoperatively. The primary outcome is 3-year RFS, and secondary outcomes are adverse events, recurrence patterns, OS, and PROs. The estimated completion date is February 2024 [63].

## 6. Conclusions

Current management of early stage EC includes surgical management with adjuvant therapy. The adjuvant treatment depends on clinical and pathological factors that assist in classifying the patients into low risk, low-intermediate, high-intermediate, and high risk groups. The molecular classification and subsequent surrogate classification using IHC and sequencing has brought along a new promising avenue by classifying EC into four molecular subgroups: POLE mutated, p53 abnormal, MMRd, and NSMP. This was a major advance in terms of characterization compared to the historical division into type 1 and type 2 EC histological subtypes. The four groups of EC currently assist with a better understanding of the risk of recurrence, and represent a promising clinical tool to assist in decision-making regarding adjuvant treatment. Specifically, POLE-mutation has low risk of recurrence and will likely transition to no adjuvant treatment required in the early stages. At the opposite end, p53 abnormal tumors have a high risk of recurrence and decreased survival and will lead to a re-classification into a higher risk group requiring chemotherapy. Clinical trials such as PORTEC-4a, RAINBO, CANSTAMP, and TAPER have the potential to provide key information for tailoring EC treatments based on molecular and clinicopathological criteria, thus leading to more optimized and personalized patient care.

## Figures and Tables

**Table 1:** Classification of endometrial cancer.

	Low Risk	Low Intermediate Risk	High Intermediate Risk	High Risk
Classification Criteria	Grade 1 or 2 <½ myometrium	Intermediate risk EC not meeting criteria for high intermediate	Definition (GOG-99): Age ≥ 70 and 1 risk factor * 50–69 yo and 2 risk factors * <50 and 3 risk factors * Definition (PORTEC) 2 out of 3: Age >60 ½ myometrium Grade 3 endometrioid FIGO = 2 RF [1]: Age > 60 ½ myometrium LVSI Serous/clear cell histology Grade 3 endometrioid	High-risk histology (serous, clear cell) Grade 3, >½ myometrial invasion, LVSI involvement (FIGO) Stage II, III/IV disease
ESGO: Risk stratification including molecular classification	Stage I-II POLEmut Stage IA MMRd/ NSMP, grade 1–2, LVSI negative	Stage IB MMRd/ NSMP grade 1–2, LVSI negative Stage IA MMRd/ NSMP grade 3, LVSI negative Stage IA p53abn and/or non-endometrioid histology without myometrial invasion	Stage I MMRd/ NSMP+ LVSI Stage IB MMRd/ NSP grade 3 Stage II MMRd/ NSMP	Stage III-IVA MMRd/ NSMP Stage I-IVA p53 abnormal with myometrial invasion Stage I-IVA MMRd/ NSMP serous, undifferentiated carcinoma, carcinosarcoma with myometrial invasion
Treatment & Supportive Evidence	Observation	Observation	Brachytherapy Supporting evidence: GOG-99 and PORTEC-1: EBRT benefits for HIR mainly PORTEC-2: vaginal brachytherapy non-inferior to EBRT	Chemotherapy + EBRT stage III Chemotherapy for high-risk histology Non-invasive high risk histology: observation vs brachytherapy vs chemotherapy Myoinvasive early stage: EBRT+ vaginal brachytherapy Supporting evidence: PORTEC-3: chemoradiation improving DFS in stage III compared to chemotherapy GOG-258 (stage III/IVA): chemoradiation and chemotherapy had similar survival; chemoradiation had less vaginal, pelvic, and para-aortic recurrences but more distant recurrences.
			GOG-249: Brachy+ 3 cycles chemotherapy vs EBRT showed no difference in survival, EBRT better pelvic control, chemotherapy more toxic	

FIGO International Federation of Gynecology and Obstetrics; GOG Gynecologic Oncology Group; PORTEC Postoperative Radiation Therapy in Endometrial Carcinoma; * GOG risk factors: >½ myometrium, Grade 2 or 3, LVSI positive.

**Table 2:** Summary of patients included in high intermediate risk and high risk EC landmark trials.

Trial Name	Year	HIR Criteria	High Risk Histology	Stage II	Stage III/IV	Treatments Compared	Survival Benefit	Recurrence Benefit
GOG-99	2004	Yes: GOG 99 definition (Table 1) *	No	Yes (occult)	No	EBRT vs. observation	No	Yes:
PORTEC-1	2000	2 out of 3: (1) age > 60 (2) >50% myometrium, (3) grade 3 histology	No	No	No	EBRT vs. observation	No	Yes:
PORTEC-2	2010	(1) Age > 60, grade1, outer 1/3 myometrium (2) Age > 60, grade 3, middle 1/3 myometrium (3) Stage IIA	No	Yes	No	Vaginal brachytherapy vs. EBRT	No	No
PORTEC-3	2018	Grade 3 with >50% and/or LVSI	Yes (stage I to III)	yes	Stage III only	Chemoradiation vs. EBRT	Yes: DFS	-
GOG-249	2019	GOG criteria: >70 years and 1 RF, 50–69 years and 2 RF, <50 years and 3 RF *	Yes	Yes	No	Brachytherapy + 3 cycles chemotherapy vs. EBRT	No	Yes: less nodal recurrence with EBRT
GOG-258	2019	No	No	No	Yes: III, IVA	Chemoradiation vs. chemotherapy	No	Yes: less local nodal recurrence, more distant recurrence with chemoradiation

* age ≥70 and one risk factor; 50–69 and two risk factors; <50 and three risk factors; GOG risk factors: >½ myometrium, Grades 2 or 3, LVSI positive.

**Table 3:** Ongoing trials for endometrial cancer (EC) treatment based on molecular classification.

Trial Name	Start Date	Estimated Completion Date	Country	Phase	Trial Type	Included EC Patients	Mutation	Treatment	Primary Outcome
**PORTEC-4a** **NCT03469674**	June 2016	Dec. 2025	EU	III	Randomized 2:1	HIR *	POLE CTNNB1 MMR TP53 L1CAM	(1) Vaginal brachytherapy (2) Experimental group: observation, brachytherapy, or EBRT	5-year vaginal recurrence
**RAINBO**	-	-	EU, USA, Canada, NZ, Australia	III	Non-randomized		POLE MMRd P53 NSMP	(1) P53: chemoradiation + −PARPi (2) MMRd: radiation + −checkpoint inhibitor (3) NSMP: Chemoradiation vs radiation + hormonal treatment (4) POLE: no adjuvant	5-year RFS
**TAPER** **NCT04705649**	July 2020	Dec. 2023	Canada	II, III	Single arm	Early stage EC	POLE NSMP	Observation	3-year pelvic recurrences (including vaginal)
**CAN-STAMP** **NCT04159155**	Nov. 2020	Sep. 2025	Canada	II, III	Randomized	Early & late stage	P53 serous	Early stage: (1): Chemotherapy + chemoradiation (2) Chemotherapy Late stage: (1): Chemotherapy (2): Chemotherapy + Niraparib	3-year RFS
**NRG-GY018** **NCT03914612** **Pembrolizumab**	July 2019	June 2023	Canada USA	III	Randomized	Stage III-IV, recurrent	MMRd	(1) Chemotherapy + placebo (2) Chemotherapy + Pembrolizumab	5-year PFS
**NRG-GY020** **NCT04214067** **Pembrolizumab**	Feb. 2020	Feb. 2024	USA Puerto Rico	III	Randomized Open label Two group	Stage I-II, HIR **	MMRd	(1) Radiation + placebo (2) Radiation + Pembrolizumab	3-year RFS

EU European Union; HIR high-intermediate risk; CTNNB1 catenin beta 1; L1CAM L1 cell adhesion molecule; MMRd mismatch repair deficient; EBRT external beam radiation therapy; NZ New Zealand p53wt p53 wild type; NSMP no specific molecular profile; RFS recurrent free survival; PFS progression free survival; * Stage IA, grade 3; stage IB, grade ½ and age >60; stage IB grade ½ and LVSI; stage IB grade 3 without LVSI; stage II microscopic, grade 1; ** GOG criteria for HIR: age > = 70 and one risk factor; 50–69 and two risk factors; <=50 and three risk factors; risk factors: LVSI, grade 3, >50% myometrial involvement.

**Table 4:** Study groups of the RAINBO trial [60].

Molecular Category	Stages	Randomized	Treatment
P53abn	All stages	Yes	(1) Chemoradiation (2) Chemoradiation+ PARP inhibitor
MMRd	II/III	Yes	(1) Radiotherapy (2) Radiotherapy and checkpoint inhibitor
NSMP	II/III	Yes	(1) Chemoradiation (2) Radiotherapy with hormonal treatment
POLEmut	All stages	No	No adjuvant treatment

p53abn p53 abnormal, MMRd mismatch repair deficiency, NSMP no specific molecular profile, POLEmut polymerase epsilon mutated.
